# Efficacy and safety of ruxolitinib in adult patients with refractory rheumatic disease-associated macrophage activation syndrome

**DOI:** 10.3389/fimmu.2025.1604648

**Published:** 2025-06-26

**Authors:** Jingjing Li, Ran Wang, Jie Chen, Antao Xu, Yakai Fu, Yanwei Lin, Xiaodong Wang, Shuang Ye, Ye Yuan, Fang Du, Qiong Fu

**Affiliations:** ^1^ Department of Rheumatology, Renji Hospital, Shanghai Jiao Tong University School of Medicine, Shanghai, China; ^2^ State Key Laboratory of Biopharmaceutical Preparation and Delivery, Institute of Process Engineering, Chinese Academy of Sciences, Beijing, China; ^3^ Shanghai Immune Therapy Institute, Shanghai, China

**Keywords:** refractory macrophage activation syndrome (MAS), rheumatic diseases, ruxolitinib, JAK inhibitor, salvage treatment, real-world evidence

## Abstract

**Objective:**

Rheumatic disease**-**associated macrophage activation syndrome (RD-MAS) is a rare and life-threatening complication of rheumatic diseases, with approximately 30% of cases being refractory to conventional therapeutic protocols. Ruxolitinib, a Janus kinase 1/2 inhibitor, has emerged as a potential therapy for refractory RD-MAS. This study aimed to evaluate its efficacy and safety in patients with refractory RD-MAS.

**Methods:**

A meticulous chart review was conducted on 20 refractory RD-MAS patients treated with ruxolitinib. Data from no ruxolitinib treatment RD-MAS patients served as historical controls. Clinical and laboratory parameters, therapeutic response, and survival outcomes were analyzed. Ruxolitinib’s efficacy and safety were evaluated, and survival rates were compared to historical controls.

**Results:**

The cohort included 20 refractory RD-MAS patients (17 females, 3 males) with underlying conditions: adult-onset Still’s disease (n = 13), systemic lupus erythematosus (n = 4), and other connective tissue diseases (CTDs) (n = 3). All patients displayed active disease at baseline. By week 8, 50% (10/20) of patients achieved partial remission, while 30% (6/20) attained complete remission. The ruxolitinib group had a significantly higher survival rate (19/20, 95%) compared to historical controls (13/21, 62%) (P = 0.011). By week 8, the median daily glucocorticoid dose dropped from 2.7 mg/kg to 0.5 mg/kg. Cytomegalovirus infection occurred in 20% (4/20) of patients.

**Conclusion:**

Ruxolitinib demonstrated substantial efficacy and tolerability in refractory RD-MAS, improving clinical outcomes and reducing glucocorticoid dependence. Although limited by its retrospective nature and small cohort size, this study suggests that ruxolitinib may serve as a potential therapy for refractory RD-MAS, warranting further investigation.

## Highlights

In a series of twenty patients with refractory RD-MAS, ruxolitinib exhibited substantial efficacy and tolerability.

## Introduction

Rheumatic disease**-**associated macrophage activation syndrome (RD-MAS) is a rare and potentially life-threatening complication of rheumatic diseases, particularly in systemic juvenile idiopathic arthritis (sJIA), systemic lupus erythematosus (SLE), and adult-onset Still’s disease (AOSD) (1). This syndrome is characterized by dysregulated immune activation and hyperinflammation (2, 3). If left untreated, it can result in extensive tissue damage and fatal outcomes (3, 4).

Currently, standardized therapeutic guidelines for adult RD-MAS are lacking, with management strategies predominantly derived from pediatric hemophagocytic lymphohistiocytosis (HLH) and sJIA-associated macrophage activation syndrome (MAS) protocols, as well as retrospective case series and case reports (4). The HLH-94 and HLH-2004 protocols were developed for primary HLH and include etoposide, dexamethasone, and cyclosporine (5, 6). Although the HLH-94 and HLH-2004 protocols are widely recognized treatment regimens for primary HLH and severe secondary HLH, their use in adult MAS—particularly those associated with rheumatic diseases—is limited due to high toxicity and differences in underlying pathophysiology (7). Furthermore, some patients failed to achieve adequate disease control with these regimens, necessitating alternative therapeutic strategies (4).

Targeted cytokine inhibition has emerged as a potential therapeutic approach for glucocorticoid- and cyclosporine-refractory MAS (2). Biologic agents, including anakinra, tocilizumab, and emapalumab, have demonstrated therapeutic potential in case reports and small-scale clinical studies (2, 8–10). However, their efficacy remains to be validated in larger cohorts (11). Notably, anakinra and tocilizumab have been implicated in modifying the clinical presentation of MAS, potentially complicating timely diagnosis (10, 12).

Ruxolitinib (RUX), a Janus kinase 1/2 (JAK1/2) inhibitor has emerged as a promising therapeutic candidate for MAS, by targeting the JAK1/2-STAT1 signaling pathway, effectively suppressing IFN-γ and other proinflammatory cytokines implicated in MAS pathogenesis (13). Currently, RUX is approved for the treatment of primary myelofibrosis, polycythemia vera, and chronic graft-versus-host disease (13). Preliminary evidence from case reports suggests that RUX may provide clinical benefits in MAS patients refractory to conventional therapies or experience disease relapse (13, 14). However, its efficacy and safety in this context remain to be systematically evaluated.

In this study, we present a cohort of patients with confirmed refractory RD-MAS. All patients received salvage therapy with ruxolitinib. To contextualize treatment outcomes, we also collected clinical data from a historical control group of patients who were managed with conventional therapies. By comparing clinical responses and adverse event profiles between these cohorts, we aim to evaluate the efficacy and safety of RUX in refractory RD-MAS, providing evidence to guide clinical decision-making and inform future therapeutic strategies.

## Methods

### Patients

Consecutive patients diagnosed with refractory RD-MAS were included from January 2022 to July 2024 at the Department of Rheumatology and Immunology, Renji Hospital, Shanghai Jiao Tong University School of Medicine, Shanghai, China. The study protocol was approved by the Ethics Committee of Renji Hospital and adhered to the ethical principles of the 1964 Declaration of Helsinki.

The inclusion criteria of this study were as follows: (1) a confirmed diagnosis of rheumatic diseases; (2) age ≥ 18 years; (3) fulfillment of the diagnostic criteria for MAS; (4) failure to achieve at least partial remission after two weeks of high-dose glucocorticoid (≥2 mg/kg/day of prednisone equivalent) or after at least three consecutive days of intravenous pulse therapy, or recurrence of clinical symptoms or laboratory abnormalities during glucocorticoid tapering; (5) administration of ruxolitinib as a salvage treatment strategy. MAS diagnosis was based on at least one of the following criteria: (1) ≥5 of the 8 HLH-2004 criteria, including persistent fever, splenomegaly, bicytopenia (hemoglobin <90 g/L, platelet count <100,000/µL, absolute neutrophil count <1,000/µL), hypertriglyceridemia or hypofibrinogenemia, elevated serum ferritin (≥3000 µg/L), increased soluble IL-2 receptor levels (>2,400 U/mL), reduced or absent natural killer cell cytotoxicity, or histopathologic evidence of hemophagocytosis; or (2) an H-score ≥ 169 points (15). Patients with tumors, central nervous system infections, or pregnancy were excluded from the study.

Additionally, a separate cohort of patients diagnosed with refractory RD-MAS between January 2017 and December 2019 who did not receive ruxolitinib were included as historical controls. The rarity and severity of RD-MAS has made it extremely hard to conduct a controlled study, therefore, has led us to use historical controls in the current study.

### Data collection

Medical records were reviewed by three independent rheumatologists. Data extraction included demographic characteristics, clinical presentation (e.g., fever, rash, lymphadenopathy, organomegaly), laboratory parameters (e.g., complete blood count, erythrocyte sedimentation rate [ESR], C-reactive protein [CRP], aspartate aminotransferase [AST], alanine aminotransferase [ALT], serum ferritin [SF], lactate dehydrogenase [LDH], triglyceride [TG], fibrinogen). Additional diagnostic assessments, including bone marrow biopsy, ultrasound, and computed tomography, were reviewed when available. Pharmacologic interventions were meticulously documented, including the administration of glucocorticoids, immunosuppressive agents, and intravenous immunoglobulin (IVIG). Baseline characteristics at refractory RD-MAS diagnosis and follow-up assessments (weeks 1, 2, 3, 8) were analyzed, as well as 8-week survival rate following the diagnosis of refractory RD-MAS. The safety and tolerability profile of ruxolitinib were systematically evaluated.

### Statistical analysis

Data from the study cohort were analyzed to identify statistically significant differences in demographic and clinical characteristics between groups. Statistical analyses were conducted using GraphPad Prism version 10.2.1 (GraphPad Software, San Diego, CA, USA). Continuous variables not normally distributed were reported as median (range); categorical variables were presented as absolute numbers and percentages. A p < 0.05 was considered significant.

### Assessment of ruxolitinib efficacy

Ruxolitinib efficacy was assessed using established criteria, incorporating clinical and laboratory parameters, including serum soluble CD25 (sCD25), SF, TG, hemoglobin, neutrophil and platelet counts, ALT levels, level of consciousness (evaluated in patients with central nervous system involvement in HLH), and hemophagocytosis in pathological specimens (16). Treatment response was classified as follows: (1) complete remission (CR), defined as normalization of all parameters; (2) partial remission (PR), defined as improvement of at least two parameters or symptoms by ≥25%, as determined by the attending physician, with specific thresholds: a ≥25% reduction in sCD25, SF, and TG; absence of blood transfusion dependence; an increase of ≥100% in neutrophil count if the baseline was <0.5 × 10^9^/L, or an increase of ≥100% returning to normal if baseline neutrophils were 0.5–2.0 × 10^9^/L; and a ≥50% reduction in ALT levels for patients with ALT >400 U/L; and (3) no response (NR), defined as failure to meet the aforementioned criteria for CR or PR (16). The overall response rate was the proportion of patients achieving CR or PR. Patients who achieved PR or CR proceeded to the long-term follow-up phase after the 8-week period.

### Ethics and data availability

This study was approved by the Ethics Committee of Renji Hospital. Informed consent was obtained from all participants for retrospective chart review, and the study was performed in accordance with the ethical standards as laid down in the 1964 Declaration of Helsinki.

## Results

### Demographics and clinical characteristics

A total of twenty patients with refractory RD-MAS, diagnosed by primary physicians due to inadequate response to standard therapy, received ruxolitinib as a salvage treatment. Demographic and baseline clinical characteristics are summarized in **Table 1**. Among 20 ruxolitinib-treated patients, 85% were female (n = 17), with a median age at RD-MAS diagnosis of 37 years (range: 23–52). Underlying conditions were primarily AOSD (n = 13), SLE (n = 4), and other connective tissue diseases (CTDs) (n = 3). All patients presented with fever at the time of diagnosis. Splenomegaly was observed in 30% (6/20) of cases, while bone marrow hemophagocytosis was identified in 45% (9/20). Hematologic parameters revealed that 8 patients (40%) exhibited moderate anemia (median hemoglobin: 94 g/L; range: 61–129), while 6 patients (30%) had severe thrombocytopenia (median platelet count: 115 × 10^9^/L; range: 25–359). The median leukocyte count was 3.69 × 10^9^/L (range: 0.58–34.03), with 7 patients (35%) experiencing severe neutropenia (median absolute neutrophil count: 1.78 × 10^9^/L; range: 0.01–32.70). Serum ferritin levels were markedly elevated across all patients (median: 5,982 ng/mL; range: 712–57,144). Hypofibrinogenemia (<2.00 g/L) was documented in 50% (n = 10) of patients (median fibrinogen: 2.00 g/L; range: 0.00–4.47). Additionally, ALT, AST, and LDH levels were elevated in the majority of cases. Prior to ruxolitinib treatment, 9 patients received immunosuppressive therapies, including etoposide (n = 5, 25%), tocilizumab (n = 4, 20%), and cyclosporine (n = 3, 15%). IVIG was also administered to 8 patients (40%). An additional 21 patients with refractory RD-MAS who did not receive ruxolitinib treatment served as historical controls. Baseline characteristics were largely comparable between the ruxolitinib-treated cohort and the historical control group, except for significantly higher serum ferritin levels and lower triglyceride levels in the ruxolitinib group (**Table 1**). Patients in the ruxolitinib group had higher baseline ferritin levels, which likely reflects more severe disease at treatment initiation compared to historical controls. This is consistent with real-world clinical practice, where ruxolitinib was generally reserved for more severe or refractory cases. As for triglyceride levels, the baseline difference was mainly due to missing data in the historical control group. Therefore, patients in the ruxolitinib group might have more severe disease compared to the historical controls, but still showed better survival outcome, which should not confound the current outcome results.

**Table 1:** Baseline demographics and clinical characteristics of patients.

Demographic	Ruxo^*^ group (N=20)	Control group (N=21)	P Value
Age, years, median (range)	37 (18-71)	29 (18-65)	0.628
Sex, female, n (%)	17 (85)	16 (76)	0.751
Death, n (%)	1 (5)	8 (38)	0.029
Previous therapies^†^			
High-dose intravenous glucocorticoids, n (%)	20 (100)	21 (100)	1.000
Average daily dose during week-1, mg/d prednisone-equivalent, median (range)	133 (100-267)	134 (100-625)	0.472
IVIG^*^, n (%)	8 (40)	5 (24)	
Etoposide, n (%)	5 (25)	8 (38)	
Tocilizumab, n (%)	4 (20)	0	
Tacrolimus, n (%)	0	1 (5)	
Ciclosporin, n (%)	3 (15)	5 (24)	
Etiology^*^			
AOSD, n (%)	13(65)	17 (81)	
SLE, n (%)	4 (20)	4 (19)	
Other CTDs, n (%)	3 (15)	0	
Fever, n (%)	20 (100)	21(100)	1.000
Splenomegaly, n (%)	6 (30)	12 (57)	0.080
Bone marrow hemophagocytosis, n (%)	9 (45)	9 (43)	0.890
Leucocyte count (10^9^/L), median (range)	3.69 (0.58-34.03)	3.15 (0.70-31.18)	0.812
Neutrophil count (10^9^/L), median (range)	1.78 (0.01-32.70)	2.30 (0.01-20.27)	0.694
Hemoglobin (g/L), median (range)	94 (61-129)	104 (63-175)	0.274
Platelet count (10^9^/L), median (range)	115 (25-359)	83 (4-188)	0.133
Aspartate aminotransferase, U/L, median (range)	72 (17-1988)	125 (28-980)	0.342
Alanine aminotransferase, U/L, median (range)	168 (10-1698)	174 (23-857)	0.944
Lactate dehydrogenase, U/L, median (range)	521 (283-10510)	794 (176-3139)	0.076
Fibrinogen, g/L, median (range)	2.00 (0.00-4.47)	1.48 (0.58-3.70)	0.123
Triglyceride concentration (mmol/L), median (range)	1.49 (0.80-17.90)	2.68 (0.85-6.35)	0.003
Serum ferritin, ng/mL, median (range)	5982 (712-57144)	1500 (1305-15000)	<0.001

^*^Ruxo, ruxolitinib; IVIG, intravenous immunoglobulin; AOSD, adult-onset Still’s disease; SLE, systemic lupus erythematosus; CTDs, connective tissue diseases.

^†^Two patients received etoposide and ciclosporin, concomitantly; One patient received combination therapy with etoposide and tocilizumab.

### Survival

**Figure 1:**
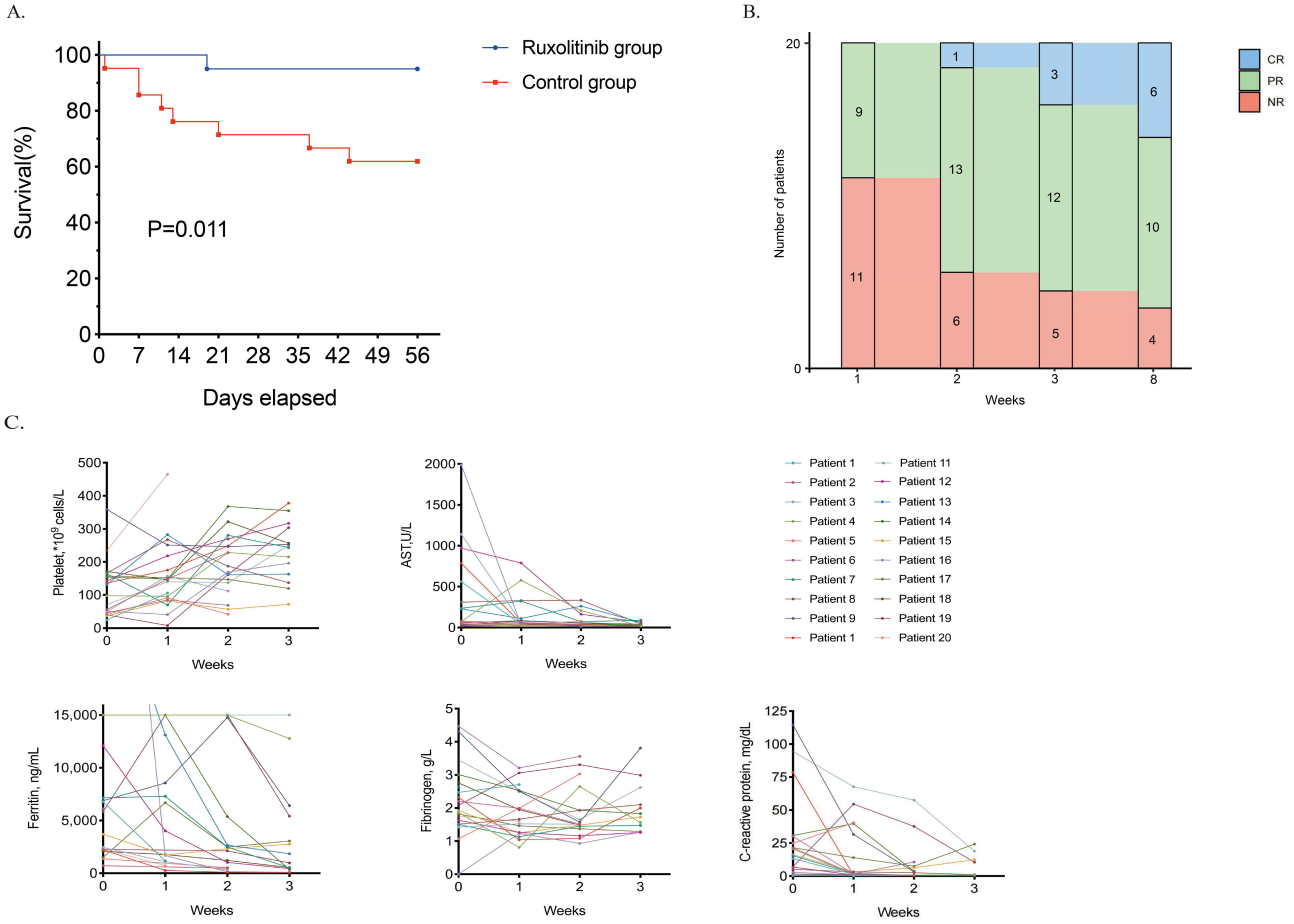
Outcomes of ruxolitinib therapy. **(A)**. Survival curve of the ruxolitinib group and the historical control group. **(B)**. The response of patients with refractory RD-MAS during the ruxolitinib treatment. **(C)**. Dynamics of RD-MAS features during the ruxolitinib treatment in study cohort. *CR, complete remission; PR, partial remission; NR, no response; AST, aspartate aminotransferase; ALT, alanine aminotransferase; TG, triglyceride concentration; RD-MAS, rheumatic disease-associated macrophage activation syndrome.

At the end of the 8-week period, 19 of 20 (95%) ruxolitinib-treated patients remained alive, demonstrating a significantly higher 56-day survival rate than historical controls (13/21, 62%; P = 0.011, **Figure 1A**).

### Response and safety of ruxolitinib

Patients in the ruxolitinib cohort received oral ruxolitinib at a dose of 5–10 mg twice daily, titrated by body weight. **Figure 1B** depicts the efficacy dynamics of ruxolitinib in refractory RD-MAS patients over 8 weeks. At week 1, 9 (45%) patients had PR. By Week 2, PR increased (n= 13), with one achieving CR. Week 3 showed further improvement (CR= 3, PR= 12, NR= 5). By week 8, 50% (10/20) and 30% (6/20) of ruxolitinib-treated patients had achieved PR and CR, respectively. The overall response rate was 80%. Four patients exhibited no response, including the patient who died of complications. One patient discontinued ruxolitinib because of challenges in tapering glucocorticoids and switched to VP therapy. The remaining two patients exhibited amelioration in laboratory indices and clinical manifestations but failed to satisfy the criteria for PR.

Ruxolitinib therapy led to rapid and significant clinical and laboratory parameter improvements (**Figure 1C**). Notably, platelet counts, AST, SF, and fibrinogen levels improved markedly over time, and CRP declined markedly in most evaluable patients. Among patients responding to ruxolitinib, peak body temperature dropped markedly following treatment initiation. Splenomegaly, detected in 6 (30%) patients, resolved completely by week 8.

Ruxolitinib was discontinued in one patient after achieving CR, and no relapse was observed during the available follow-up period. Cyclosporine was initiated in 6/16 (37.5%) patients 2–4 weeks after RD-MAS achieving PR/CR to optimize control of the underlying disease. All patients who achieved remission (PR/CR) underwent long-term follow-up. As of December 2024, all 16 remained clinically stable, with no additional deaths reported. The duration of ruxolitinib treatment varied among patients and was individualized based on clinical response, with a median duration of 3 months.

Adverse events (AEs) were minimal and predominantly mild. Cytomegalovirus (CMV) infections occurred in four patients (20%), all classified as Grade 1 or 2. No severe AEs, including serious infections or thrombotic events, were reported during treatment.

### Glucocorticoid-sparing effect

**Figure 2:**
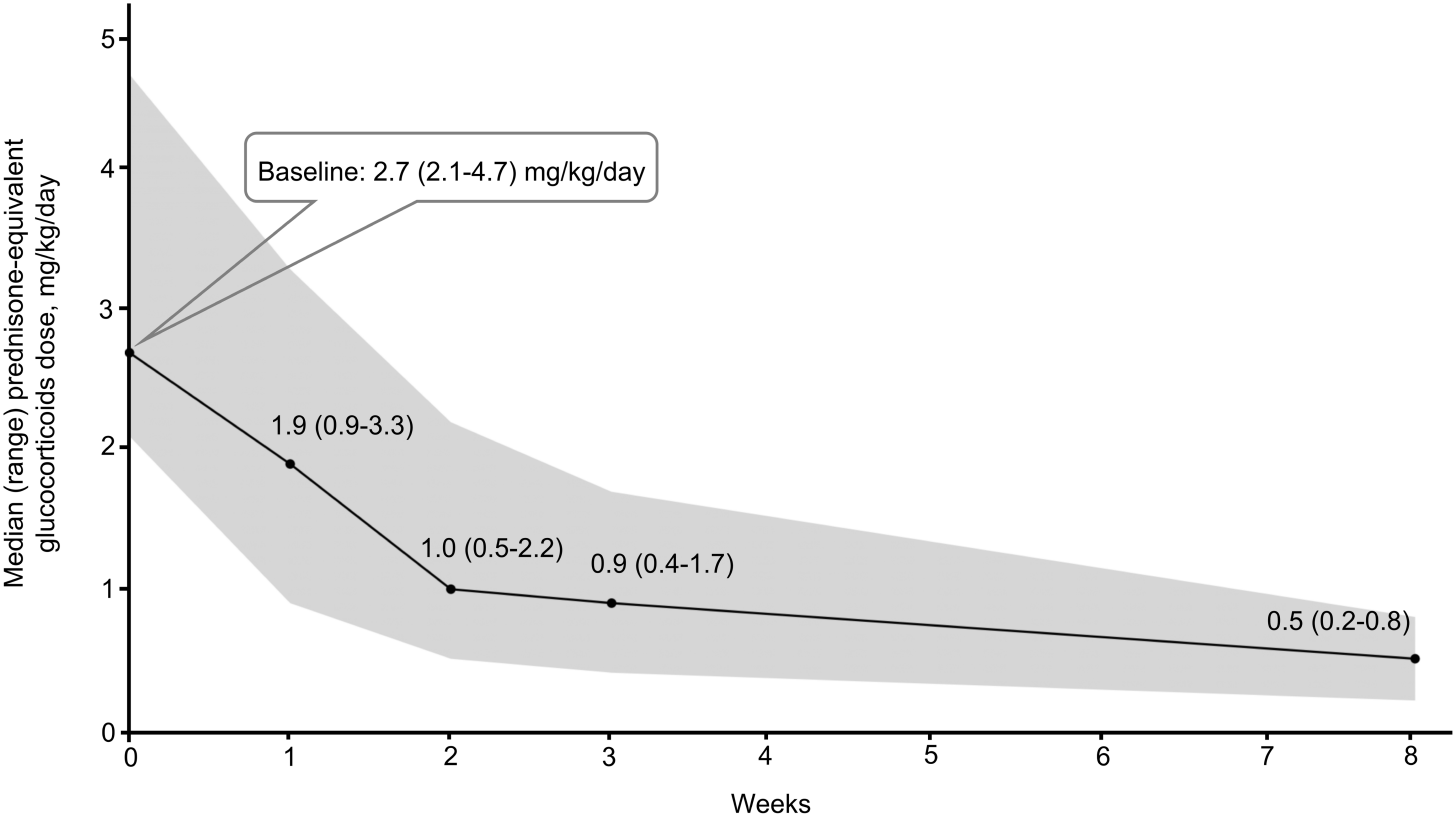
Glucocorticoid tapering in the ruxolitinib cohort.

All patients received high-dose glucocorticoids when starting ruxolitinib therapy. As illustrated in **Figure 2**, the initial median glucocorticoid dose (prednisone equivalent) was 2.7 mg/kg/day (range: 2.1– 4.7). Over 8 weeks, the median dose decreased by 81%, from 2.7 mg/kg/day in the week prior to ruxolitinib initiation to 0.5 mg/kg/day (range: 0.2– 0.8) at week 8.

## Discussion

Rheumatic disease**-**associated macrophage activation syndrome (RD-MAS) is a life-threatening complication of rheumatologic diseases, characterized by uncontrolled activation of T cells and macrophages and a subsequent surge in proinflammatory cytokines, including interleukin (IL)-1, IL-6, and interferon-γ (IFN-γ) (2, 3). Currently, no universally accepted salvage regimen exists for RD-MAS, and the literature is limited to case reports and small-scale studies evaluating therapies following first-line treatment failure. Therefore, identifying an effective salvage regimen is crucial for improving outcomes in refractory RD-MAS. Although biologics targeting individual cytokines, such as IL-1 or IL-6, have shown therapeutic efficacy, their effects are confined to specific inflammatory pathways. Notably, agents like anakinra and tocilizumab may alter the clinical manifestations of RD-MAS, potentially delaying its recognition and diagnosis (10, 12). Ruxolitinib, an oral JAK1/2 inhibitor, exerts a broader immunomodulatory effect by blocking multiple cytokine signaling cascades simultaneously (17). This wider inhibition of pro-inflammatory pathways may underlie its superior therapeutic potential in controlling the cytokine storm characteristic of RD-MAS. Ruxolitinib has garnered considerable attention, with emerging case reports indicating its therapeutic potential in refractory or relapsed RD-MAS (13, 18). A recent paper published in Blood also provided evidence supporting the clinical efficacy of ruxolitinib in refractory MAS by inhibiting JAK-STAT signaling to modulate neutrophil activation (19).

Efficacy assessments were performed weekly during the initial three weeks of ruxolitinib therapy. Most clinical and laboratory abnormalities improved within one week of ruxolitinib initiation. Notably, the earliest PR was observed by day 3, indicating a rapid response in refractory RD-MAS. By week 8, the overall response rate was 80% (16/20), comprising a CR in 30% (6/20) and PR in 50% (10/20) of cases. Among patients achieving PR, nearly all aberrant laboratory parameters normalized, except for serum ferritin. The delayed normalization of ferritin levels is primarily attributed to its biological properties, the complexity of iron metabolism, and the lag in therapeutic response (20–22).

A key clinical benefit of ruxolitinib therapy is the high survival rate in refractory RD-MAS. While survival outcomes in this population remain inadequately characterized in the literature, clinical experience suggests that individuals who fail to achieve an adequate response to conventional therapies face poor prognoses (3). In this study, ruxolitinib achieved an 80% survival rate. One patient died on day 19 due to multiple organ dysfunction, including hepatic and cardiac failure and lower gastrointestinal hemorrhage. Given the severity of the underlying disease and pre-existing organ failure prior to ruxolitinib initiation, direct causality remains uncertain. During long-term follow-up of the 16 patients who achieved remission (PR/CR), no new mortality events were observed. These findings suggest that ruxolitinib may provide sustained clinical benefit and favorable long-term efficacy.

Furthermore, this study demonstrated a significant reduction in median daily glucocorticoid dosage following ruxolitinib administration, decreasing from 2.7 mg/kg to 0.5 mg/kg.

The safety profile of ruxolitinib observed in this study was favorable, with cytomegalovirus (CMV) infection occurring in 20% (4/20) of patients. Notably, no severe adverse events, including serious infections or thrombotic complications, were reported.

This study has several limitations, including its retrospective nature, small sample size, and single-center design. As a retrospective analysis, data collection was based on previously recorded medical records, which may introduce information bias and selection bias. Although a historical control group was included to enhance the rigor of comparison, the absence of prospective randomization may still allow for potential confounding factors, limiting causal inference regarding the efficacy of ruxolitinib. The use of historical controls also introduces certain limitations, such as potential temporal biases related to changes in supportive care, diagnostic criteria, and data completeness over time. Additionally, the patient cohort comprised various rheumatic diseases, including AOSD, SLE, and other CTDs, which are known to differ in their immunopathological features. Some patients had received prior treatments such as tocilizumab, cyclosporine, or etoposide before ruxolitinib. Although these may affect treatment response, the small sample size limited further subgroup analysis. This potential confounder should be addressed in future studies. Moreover, this was a single-center study involving primarily Han Chinese patients, which may limit the generalizability of the findings to other populations and settings. Future prospective studies with larger cohorts in more diverse populations are warranted to further elucidate the effectiveness and safety profile of ruxolitinib in refractory RD-MAS.

In summary, this study provides additional evidence supporting the effectiveness and safety of ruxolitinib in refractory RD-MAS. This agent demonstrates a notable capacity to improve clinical symptoms and laboratory markers over the long term, while significantly reducing glucocorticoid dependence and maintaining an overall favorable safety profile.

## Data Availability

The raw data supporting the conclusions of this article will be made available by the authors, without undue reservation.
